# Chemotherapy receipt in affected *BRCA1*/2 and *PALB2* carriers with operable breast cancer: the impact of early detection and pre-diagnostic awareness on clinical outcomes and treatment

**DOI:** 10.1186/s13053-025-00314-x

**Published:** 2025-04-24

**Authors:** Stephanie M. Wong, Carla Apostolova, Amina Ferroum, Basmah Alhassan, Ipshita Prakash, Mark Basik, Karyne Martel, Sarkis Meterissian, David Fleiszer, Nora Wong, Michaela Bercovitch Sadinsky, Talia Malagon, Jean Francois Boileau, William D. Foulkes

**Affiliations:** 1 Department of Surgery, McGill University, Montreal, QC Canada; 2 Stroll Cancer Prevention Centre, Jewish General Hospital, Montreal, QC Canada; 3 Department of Oncology, McGill University, Montreal, QC Canada; 4 Lady Davis Institute for Medical Research, Jewish General Hospital, Montreal, QC Canada; 5 Department of Human Genetics, McGill University, Montreal, QC Canada; 6 St Mary’s Research Centre, Montreal West Island Integrated University Health and Social Services Centre, Montreal, QC Canada; 7 Segal Cancer Centre, Jewish General Hospital, 3755 Cote Ste Catherine, E713, Montreal, QC H3T1E2 Canada

**Keywords:** Breast neoplasms, *BRCA1/2*, Hereditary breast cancer, Chemotherapy

## Abstract

**Purpose:**

While enhanced breast screening of germline pathogenic variant (GPV) carriers results in earlier stage at diagnosis, the impact of tumour biology and GPV on chemotherapy receipt in early-stage disease remains understudied.

**Methods:**

We retrospectively reviewed treatment administered following a first diagnosis of *BRCA1/2*- and *PALB2*-associated breast cancer between 2002 and 2022. Chemotherapy receipt was compared according to tumor size, biologic subtype, and GPV. Subgroup analyses were performed in women with T1N0 disease and in those with pre-diagnostic awareness of their GPV.

**Results:**

Overall, 309 affected *BRCA1/2* and *PALB2* carriers with a median age of 43 years at breast cancer diagnosis (range, 19–80 years) were included; 160 (51.8%) *BRCA1*, 130 (42.1%) *BRCA2*, and 19 (6.1%) *PALB2* carriers. Chemotherapy was administered in 70.9% of index breast cancer cases and was significantly associated with younger age, tumor size, histologic grade, nodal status, and biologic subtype (all *p* < 0.05). Chemotherapy receipt was 80.6% in *BRCA1*-associated breast cancers compared to 56.9% in *BRCA2* and 84.2% in *PALB2* associated breast cancers (*p* < 0.001). In subgroup analysis of early stage, T1N0 disease, chemotherapy was administered in 78.9% *BRCA1* and 59.5% *BRCA2/PALB2* patients (*p* = 0.04). Pre-diagnostic awareness of a GPV in *BRCA1/2* or *PALB2* was associated with smaller invasive tumors (%T1, 50% vs. 32.9%; *p* = 0.002) and node-negative invasive disease (87.1% vs. 72.2%), as well as a reduced likelihood of chemotherapy (59.7% vs. 74.3%, *p* = 0.02).

**Conclusion:**

Chemotherapy receipt is high in *BRCA1/2* and *PALB2*-associated breast cancers including in early stage, node-negative disease. Pre-diagnostic awareness is associated with a lower likelihood of requiring chemotherapy for a breast cancer diagnosis.

**Supplementary Information:**

The online version contains supplementary material available at 10.1186/s13053-025-00314-x.

## Introduction

While enhanced surveillance of germline *BRCA1/2* and *PALB2* carriers results in earlier stage at breast cancer diagnosis and improved outcomes [1, 2], the impact of tumor biology and specific germline pathogenic variant (historically termed ‘mutation’) on chemotherapy receipt in early-stage disease remains understudied. Approximately 72% of *BRCA1* carriers and 22–34% of *BRCA2* and *PALB2* carriers diagnosed with breast cancer present with triple-negative breast cancer (TNBC) [3, 4] a particularly aggressive biologic subtype that almost always necessitates chemotherapy even if tumors are small at diagnosis [5]. Although the majority of *BRCA2* and *PALB2*-associated breast cancers and 20% of *BRCA1*-associated breast cancers are hormone-receptor positive, human epidermal growth factor receptor negative (ER + HER2-), germline pathogenic variant carriers tend to have highly proliferative ER + HER2- tumors with markedly elevated 21-gene recurrence scores, for which chemotherapy is also recommended [6–8]. 

Pre-diagnostic awareness of a germline pathogenic variant in *BRCA1/2* or *PALB2* allows for enhanced screening and risk-reducing mastectomy, the latter of which dramatically lowers the likelihood of being diagnosed with breast cancer [9, 10]. For patients who elect to forgo risk-reducing surgery, protocols combining breast magnetic resonance imaging (MRI) and mammographic screening aim to detect breast cancers early. In one study of 105 *BRCA1/2* germline pathogenic variant carriers diagnosed with breast cancer between 2005 and 2016, chemotherapy receipt was 59% overall, yet in the 42 patients with pre-diagnostic awareness of their *BRCA* status, 86% were diagnosed with stage 0-I disease and only 29% received chemotherapy [11]. Perhaps because of the older median age of this cohort (50.4 years), their results contrast with those from other studies of younger affected *BRCA1/2* carriers, where chemotherapy receipt has been documented in upwards of 92% of carriers [12, 13]. For *PALB2* carriers, breast cancers are felt to be biologically similar to *BRCA2* cases, however there remains a paucity of data on breast cancer treatment and outcomes [14, 15]. 

To further characterize factors associated with chemotherapy receipt and help advise unaffected *BRCA1/2* and *PALB2* carriers about the likelihood of requiring chemotherapy if diagnosed with an early-stage breast cancer in the future, we performed a multi-centered retrospective cohort study of affected *BRCA1/2* and *PALB2* carriers diagnosed between 2002 and 2022. Our primary objective was to evaluate the proportion of affected carriers who received chemotherapy for an index breast cancer diagnosis, including those with early stage, T1N0 disease. A secondary objective was to evaluate the impact of pre-diagnostic awareness on clinical and treatment related outcomes, including the need for chemotherapy and adjuvant endocrine therapy.

## Methods

### Cohort selection

**Fig. 1:**
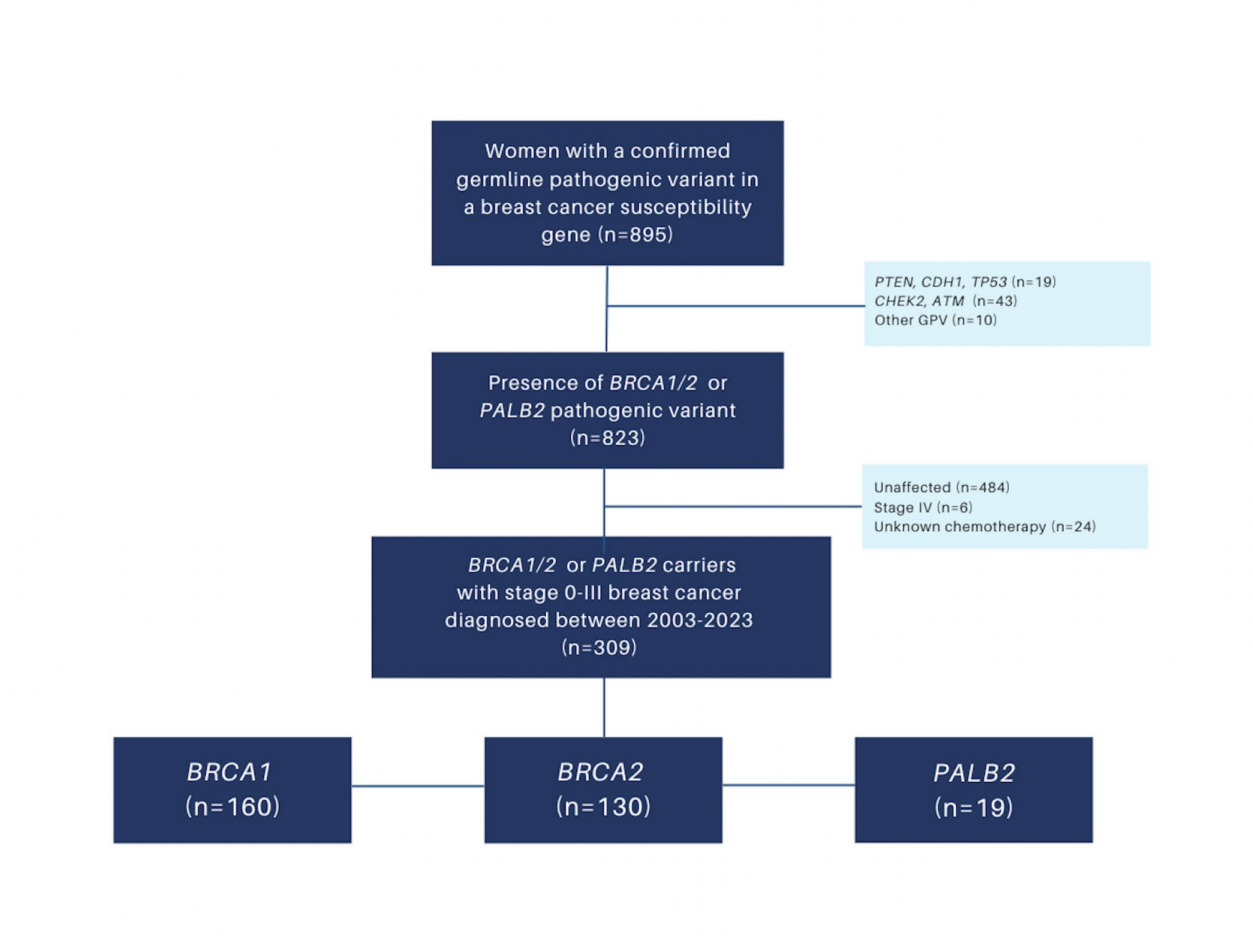
Cohort selection

Following institutional review board approvals from the Jewish General Hospital (CIUSSS West-Central Montreal Research Ethics Board, reference no. MP-05-2022-3018) and McGill University Health Centre (CIUSSS Central West of the Island of Montreal Research Ethics board), we retrospectively reviewed the medical records of female patients with a confirmed germline pathogenic variant in *BRCA1/2* or *PALB2* who were diagnosed or treated with at least one in situ or invasive stage I-III breast cancer at two McGill University-affiliated institutions in Montreal, Canada between January 2002 and January 2022 (Fig. 1). Variants were annotated according to the American College of Medical Genetics (ACMG) five-tiered categorization, and those which were pathogenic or likely pathogenic were included in the cohort, while variants of uncertain significance or any likely benign or benign variants were excluded. Patients who did not have a diagnosis of breast cancer, had *de novo* stage IV breast cancer at diagnosis, or those for whom pathology or systemic therapy details were unknown were also excluded from the cohort. Due to the retrospective nature of the study and study period, the requirement for informed consent was waived by the research ethics boards of participating centres.

### Outcomes and independent variables

The primary outcome was any chemotherapy receipt for an index diagnosis of breast cancer, including neoadjuvant or adjuvant chemotherapy. Chemotherapy was defined as any systemic therapy that included anthracycline and/or taxane and/or platinum-based regimens, with immunotherapy or HER2-directed therapy when applicable.

Independent variables of interest included germline pathogenic variant, divided into *BRCA1* vs. *BRCA2* vs. *PALB2*, age at index breast cancer diagnosis (categorized as < 30 years, 30–39 years, 40–49 years, 50–59 years, and 60 + years), family history (breast cancer, ovarian cancer, pancreatic cancer, and male breast cancer), and pre-diagnostic awareness, defined as knowledge of a germline pathogenic variant prior to breast cancer diagnosis if genetic testing had been performed at least one month or more prior to diagnostic biopsy. Clinical variables, such as tumor histology, histologic grade, biologic subtype (ER + HER2-, TNBC, or HER2+), tumor size, and nodal status were determined clinically at the time of biopsy in the setting of neoadjuvant treatment or based on final pathologic staging in the setting of primary surgery. Treatment approach (primary surgery or neoadjuvant chemotherapy), index breast surgery (defined as breast conserving surgery, unilateral mastectomy, or bilateral mastectomy), and adjuvant therapy (radiation and endocrine therapy) were also evaluated.

### Statistical analysis

All data variables were collected and managed using REDCap electronic data capture tools hosted at the Jewish General Hospital Lady Davis Institute for Medical Research [16]. Descriptive statistics were used to summarize baseline demographic and clinical characteristics of the study population. The Chi-squared and Fisher’s exact tests were used to compare categorical variables between groups, comparing clinical and treatment related variables by germline pathogenic variant as well as clinical variables associated with chemotherapy receipt. Subgroup analysis was then performed among patients with T1N0 breast cancer to evaluate the association between germline pathogenic variant, tumor biology, and chemotherapy receipt in early-stage disease. We then performed additional analyses evaluating the impact of pre-diagnostic awareness on the clinicopathologic outcomes and treatment associated with an index breast cancer diagnosis. Analyses were carried out with SAS software version 9.4 (SAS Institute Inc., Cary, NC, USA) with a *p* value of < 0.05 used to indicate statistical significance.

## Results

### Cohort characteristics

Of 895 patients with a known pathogenic variant in *BRCA1/2* and *PALB2* or other breast cancer susceptibility genes, we identified 312 affected female *BRCA1*/2 and *PALB2*-positive patients with breast cancer. After exclusion of 3 (1.0%) patients with stage IV disease, the final analytic cohort included 309 *BRCA1/2* and *PALB2*-positive women with operable breast cancer; 160 (51.8%) *BRCA1*, 130 (42.1%) *BRCA2*, and 19 (6.1%) *PALB2* carriers. Of these 309 patients, 72 (23.3%) were aware of their germline pathogenic variant prior to the development of their first breast cancer. Notably, none of the 3 excluded stage IV cases were aware of their germline pathogenic variant prior to diagnosis.

In the analytic cohort, the median age at index breast cancer diagnosis was 43 years; 40 years (range 19–72) for *BRCA1*, 44 (range, 27–80) for *BRCA2*, and 50 (range, 42–61) for *PALB2*. Clinical characteristics and treatment details by pathogenic variant are shown in Table 1. *BRCA2* carriers were more likely to be diagnosed with ductal carcinoma in situ (18.5% vs. 4.4%, *p* < 0.001) compared to *BRCA1* carriers. *BRCA1*-associated breast cancers tended to be earlier onset at diagnosis (*p* = 0.003), and when invasive, were higher grade (*p* < 0.001), node-negative (*p* = 0.01), and more commonly TNBC (%TNBC: 69.4% *BRCA1* vs. 24.7% *BRCA2* vs. 31.6% *PALB2*; *p* < 0.001). In contrast, *BRCA2* and *PALB2*-associated invasive breast cancers were more likely to be node-positive and were ER + HER2- in 63.2–70% of cases (Table 1). In terms of treatment approach, 36% of patients were treated with neoadjuvant systemic therapy, including 42.5% of *BRCA1* and 47.4% of *PALB2* cases, compared to only 26.2% of *BRCA2* patients (*p* = 0.01). Approximately half (48.4%) of patients received breast conserving therapy, while the remaining half were treated with mastectomy, including 15.8% who underwent unilateral and 34.9% who underwent bilateral mastectomy, with no difference by germline pathogenic variant (*p* = 0.31).

**Table 1:** Demographic and clinical differences between *BRCA1/2* and *PALB2* patients with breast cancer (*n* = 309)

Characteristic	*BRCA1* (*n* = 160)	*BRCA2* (*n* = 130)	*PALB2* (*n* = 19)	*P*-value	Total (*n* = 309)
Age at First Breast Cancer Diagnosis *– n*, (%)				0.003	
<30 years	22 (13.8)	6 (4.6)	1 (5.3)		29 (9.4)
30–40 years	56 (35.0)	34 (26.2)	1 (5.3)		91 (29.5)
40–50 years	36 (22.5)	48 (36.9)	7 (36.8)		91 (29.5)
50–60 years	31 (19.4)	27 (20.8)	5 (26.3)		63 (20.4)
60 + years	15 (9.4)	15 (11.5)	5 (26.3)		35 (11.3)
Family History in a 1st or 2nd degree relative – *n*, (%)					
Breast Cancer	112 (70.0)	90 (69.2)	11 (57.9)	0.94	187 (68.3)
Ovarian Cancer	56 (35.0)	24 (18.5)	3 (15.8)	0.004	72 (26.3)
Pancreatic Cancer	21 (13.1)	13 (10.0)	3 (15.8)	0.62	37 (12.0)
Male Breast Cancer	4 (2.8)	8 (7.1)	1 (5.9)	0.25	13 (4.7)
Pre-diagnostic awareness of GPV status – *n*, (%)				0.18	
Awareness of GPV prior to breast cancer diagnosis	44 (27.5)	25 (19.2)	3 (15.8)		72 (23.3)
Genetic testing after breast cancer diagnosis	116 (72.5)	105 (80.8)	16 (84.2)		237 (76.7)
Histology – *n*, (%)				< 0.001	
Ductal carcinoma in situ (DCIS)	7 (4.4)	24 (18.5)	-		31 (11.3)
Invasive ductal carcinoma	128 (80.0)	84 (64.6)	16 (84.2)		228 (73.8)
Invasive lobular carcinoma	3 (1.9)	7 (5.4)	2 (10.3)		12 (3.9)
Other/unknown	22 (13.8)	15 (11.5)	1 (5.3)		38 (12.3)
Histologic Grade* – *n*, (%)				< 0.001	
Grade I	2 (1.3)	5 (4.7)	-		7 (2.5)
Grade II	26 (17.0)	37 (34.9)	10 (52.6)		73 (26.3)
Grade III	125 (81.7)	64 (60.4)	9 (47.4)		198 (71.2)
Tumor Size* *– n*, (%)				0.09	
T1a/cT1b	21 (13.7)	17 (16.0)	2 (10.5)		40 (14.4)
T1c	38 (24.8)	27 (25.5)	5 (26.3)		70 (25.2)
T2	65 (42.5)	32 (30.2)	11 (57.9)		108 (38.9)
T3/T4	15 (9.8)	9 (8.5)	1 (5.3)		25 (9.0)
Unknown tumor size	14 (9.2)	21 (19.8)	-		35 (12.6)
Nodal Status* – *n*, (%)				0.01	
N0	124 (81.1)	76 (71.7)	10 (52.6)		210 (75.5)
N1/N2	29 (18.9)	30 (28.3)	9 (47.4)		68 (24.5)
Biologic Subtype* – *n*, (%)				< 0.001	
ER + HER2-	38 (25.9)	65 (69.9)	12 (63.2)		115 (44.4)
HER2+	7 (4.8)	5 (5.4)	1 (5.3)		13 (5.0)
TNBC	102 (69.4)	23 (24.7)	6 (31.6)		131 (50.6)
Treatment Approach – *n*, (%)				0.01	
Primary/upfront surgery	92 (57.5)	96 (73.9)	10 (52.6)		198 (64.1)
Neoadjuvant systemic therapy	68 (42.5)	34 (26.2)	9 (47.4)		111 (35.9)
Index Breast Surgery – *n*, (%)				0.31	
Breast conserving surgery	79 (50.3)	60 (46.9)	8 (42.1)		147 (48.4)
Unilateral mastectomy	18 (11.5)	26 (20.3)	4 (21.1)		48 (15.8)
Bilateral mastectomy	60 (38.2)	42 (32.8)	7 (36.8)		109 (34.9)
Adjuvant Radiotherapy – *n*, (%)				0.49	
Yes	85 (53.1)	78 (60.0)	11 (57.9)		174 (56.3)
No	75 (46.9)	52 (40.0)	8 (42.1)		135 (43.7)
Adjuvant Endocrine Therapy – *n*, (%)				< 0.001	
Yes	33 (20.6)	61 (46.9)	9 (47.4)		103 (33.3)
No	127 (79.4)	69 (53.1)	10 (52.6)		206 (66.7)
Any Chemotherapy Receipt^†^ – *n*, (%)				< 0.001	
Yes	129 (80.6)	74 (56.9)	16 (84.2)		219 (70.9)
No	31 (19.4)	56 (43.1)	3 (15.8)		90 (29.1)

*ER + HER2-* estrogen receptor positive, HER2-negative, *GPV* Germline pathogenic variant, *HER2* + HER2-positive, *TNBC* Triple negative breast cancer

*Reported for invasive breast cancer cases only

^†^Includes neoadjuvant or adjuvant chemotherapy

Chemotherapy was administered in 70.9% of index breast cancer cases diagnosed in *BRCA1/2* and *PALB2* carriers. Chemotherapy receipt varied significantly by pathogenic variant and age at diagnosis, with *BRCA1* carriers, *PALB2* carriers, and early-onset breast cancers diagnosed under 30 years of age receiving chemotherapy in greater than 80% of cases (Table 2). In contrast, only 56.9% of *BRCA2* carriers and 11.3% of patients over 60 years old received chemotherapy for an index breast cancer diagnosis. In cases of invasive breast cancer, higher histologic grade, tumor size, and node positive disease were all significantly associated with chemotherapy receipt (all *p* < 0.001). Patients with ER + HER2- invasive breast cancer received chemotherapy in 74% of cases, compared to 90.0% of TNBC and 91.7% of HER2 + invasive breast cancers (*p* = 0.006). The majority of chemotherapy received was anthracycline-based (55.0%), with or without taxanes or the addition of platinum agents.

**Table 2:** Clinical and demographic factors associated with chemotherapy receipt in *BRCA1/2* and *PALB2* carriers with operable breast cancer (*n* = 309)

Characteristic	Cohort	Proportion receiving chemotherapy	
	No. (%)	%	*p*-value
Germline Pathogenic Variant (GPV) – *n*, (%)			< 0.001
* BRCA1*	160 (51.8)	80.6	
* BRCA2*	130 (42.1)	56.9	
* PALB2*	19 (6.2)	84.2	
Age at Index Breast Cancer Diagnosis *– n*, (%)			0.040
<30 years	29 (9.4)	84.6	
30–40 years	91 (29.5)	72.4	
40–50 years	91 (29.5)	74.4	
50–60 years	63 (20.4)	54.7	
60 + years	35 (9.5)	11.3	
Biopsy Histology – *n*, (%)			< 0.001
Ductal carcinoma in situ (DCIS)	31 (10.3)	0.0	
Invasive ductal carcinoma	228 (73.8)	84.2	
Invasive lobular carcinoma	12 (3.9)	66.7	
Other/unknown	38 (12.3)	50.0	
Histologic Grade*			< 0.001
Grade I	7 (2.5)	14.3	
Grade II	73 (26.3)	69.9	
Grade III	198 (71.2)	84.3	
Biologic Subtype* – *n*, (%)			0.006
HR + HER2-	92 (43.6)	73.9	
HER2+	12 (5.7)	91.7	
TNBC	108 (50.9)	90.0	
Tumour Size* *– n*, (%)			< 0.001
cT1a-b	36 (13.0)	50.0	
cT1c	66 (23.7)	87.9	
cT2	104 (37.4)	96.2	
cT3-T4	23 (8.3)	95.7	
Unknown	49 (17.6)	42.9	
Nodal Status* – *n*, (%)			< 0.001
cN0	210 (75.5)	72.3	
cN1-N2	68 (24.5)	98.5	

*ER + HER2-* estrogen receptor positive, HER2-negative, *HER2* + HER2-positive, *TNBC* Triple negative breast cancer

*Reported for invasive breast cancer cases only

**Fig. 2:**
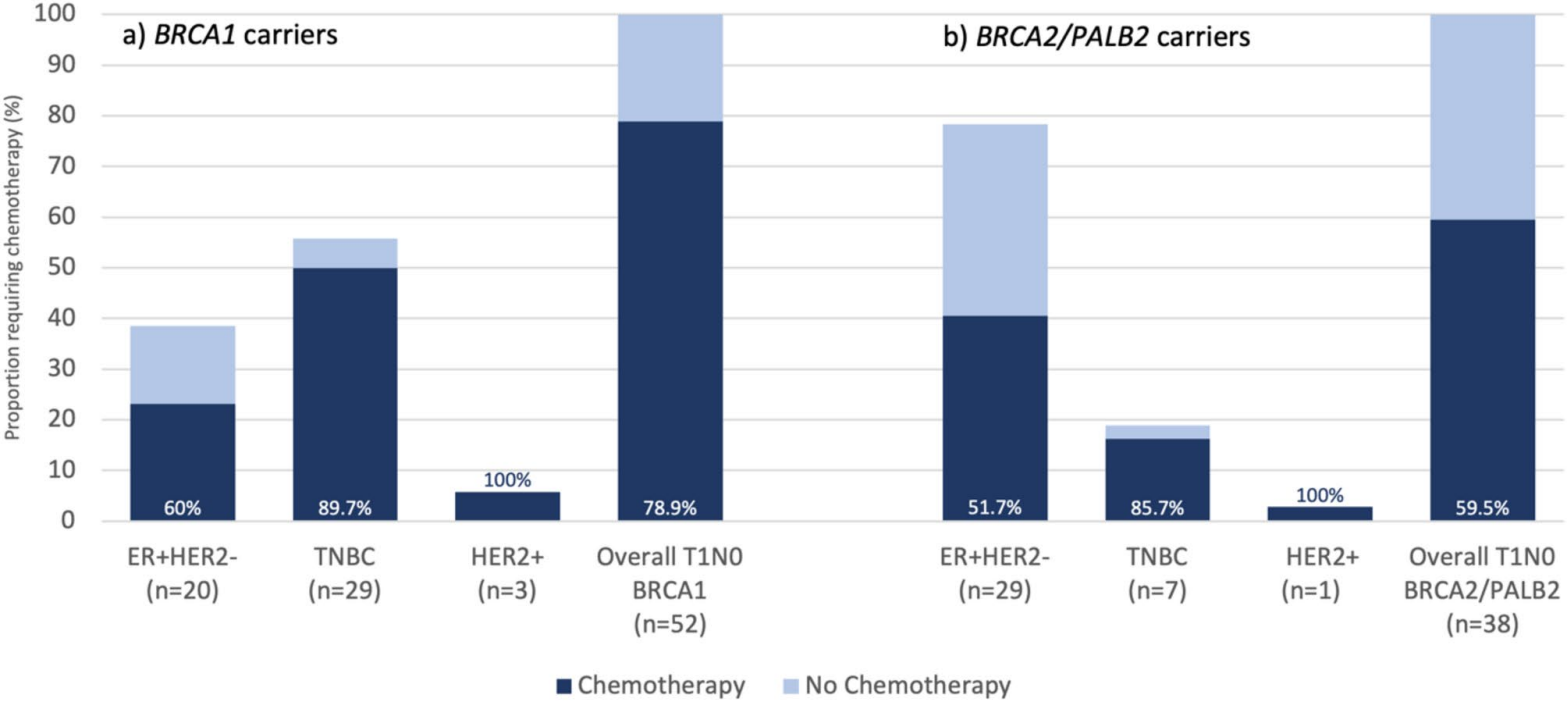
Chemotherapy receipt amongst (**a**)* BRCA1* and (**b**) *BRCA2/PALB2* carriers with T1N0 invasive breast cancer (*n*=89). *ER+HER2-* estrogen receptor positive, HER2-negative; *HER2*+ HER2-positive; *TNBC* Triple negative breast cancer

In subgroup analysis of 89 patients with T1N0 disease, 52 (58.4%) *BRCA1*, 32 (36.0%) *BRCA2*, and 5 (5.6%) *PALB2* carriers were included. In this subgroup of patients with early-stage invasive disease, chemotherapy was administered in 41 (78.9%) *BRCA1* compared to 22 (59.5%) *BRCA2*/*PALB2* patients (*p* = 0.04). This was largely driven by the increased incidence of TNBC which accounted for 56% of *BRCA1* carriers with T1N0 breast cancer (90% of whom received chemotherapy), while only 15.6% of *BRCA2* and 40% of *PALB2* carriers had T1N0 TNBC (of which 80% and 100% received chemotherapy, respectively; eTable 1). In comparison, 76% of *BRCA2/PALB2* carriers with T1N0 breast cancer had ER + HER2- disease, only half of whom received chemotherapy (Fig. 2, eTable 1). Anthracycline-based regimens (with or without taxanes or the addition of platinum agents) constituted the majority of chemotherapy received in T1N0 tumours (57.1%), followed by taxane-based regimens in (27.0%), platinum-based regimens (9.5%) and other regimens (6.4%).

### Pre-diagnostic awareness of *BRCA1/2* and *PALB2* status and treatment receipt

In the 72 (23.3%) patients who had pre-diagnostic knowledge of their *BRCA1*/2 or *PALB2* pathogenic variant, there were no significant differences in age at breast cancer diagnosis, family history of breast or pancreatic cancer, or distribution of germline pathogenic variants compared to the 237 patients who underwent testing after diagnosis (all *p* > 0.05, Table 3). Women with pre-diagnostic awareness were however more likely to have a family history of ovarian cancer than those who underwent testing after diagnosis (37.5% vs. 23.6%, *p* = 0.02) and were more likely to be enrolled in MRI screening (79.2% vs. 1.3%, *p* < 0.001). When diagnosed with breast cancer, tumor histology, histologic grade, and biologic subtype of the index breast cancer were not significantly different between the two groups (all *p* > 0.05). However, women with pre-diagnostic awareness were more likely to have MRI detected tumours (61.1% vs. 0.84%, *p* < 0.001), be diagnosed with invasive tumors less than 2 cm (50% vs. 32.9%, *p* = 0.002) and present with node negative disease (87.1% vs. 72.2%, *p* = 0.02). In contrast, patients who underwent genetic testing after diagnosis were more likely to present with a clinical abnormality/palpable mass (70.1% vs. 11.1%) or with a mammographically-detected abnormality (29.1% vs. 22.2%) relative to those with pre-diagnostic awareness (*p* < 0.001).

**Table 3:** Clinicopathologic and treatment characteristics in *BRCA1/2* and *PALB2* patients with breast cancer based on pre-diagnostic awareness of germline pathogenic variant (*n* = 309)

Characteristic	Awareness of GPV prior to BC diagnosis (*n* = 72)	Genetic testing after BC diagnosis (*n* = 237)	*P*-value
Age at First Breast Cancer Diagnosis *– n*, (%)			0.27
<30 years	5 (6.9)	24 (10.1)	
30–40 years	22 (30.6)	69 (29.1)	
40–50 years	16 (22.2)	75 (31.7)	
50–60 years	17 (23.6)	46 (19.4)	
60 + years	12 (16.7)	23 (9.7)	
Family History in a 1st or 2nd degree relative – *n*, (%)			
Breast Cancer	56 (77.8)	159 (67.1)	0.08
Ovarian Cancer	27 (37.5)	56 (23.6)	0.02
Pancreatic Cancer	5 (6.9)	32 (13.5)	0.13
Male Breast Cancer	7 (7.9)	8 (3.8)	0.17
Germline Pathogenic Variant – *n*, (%)			0.18
* BRCA1*	44 (61.1)	116 (49.0)	
* BRCA2*	25 (34.7)	105 (44.3)	
* PALB2*	3 (4.2)	16 (6.8)	
Screening MRI – *n*, (%)			< 0.001
Yes	57 (79.2)	3 (1.3)	
No	15 (20.8)	234 (98.7)	
Method of Breast Cancer Detection – *n*, (%)			< 0.001
Clinical abnormality/palpable mass	8 (11.1)	166 (70.0)	
Mammographically-detected abnormality	16 (22.2)	69 (29.1)	
MRI-detected abnormality	44 (61.1)	2 (0.84)	
Occult breast cancer on risk-reducing mastectomy	4 (5.6)	-	
Histology – *n*, (%)			0.53
Ductal carcinoma in situ (DCIS)	10 (13.9)	21 (8.9)	
Invasive ductal carcinoma	53 (73.6)	175 (73.8)	
Invasive lobular carcinoma	2 (2.8)	10 (4.2)	
Other/unknown	7 (9.7)	31 (13.1)	
Histologic Grade* – *n*, (%)			0.40
Grade I	3 (4.8)	4 (1.9)	
Grade II	15 (24.2)	58 (26.9)	
Grade III	44 (71.0)	154 (71.3)	
Tumor Size* *– n*, (%)			0.002
T1a/T1b	17 (27.4)	19 (8.8)	
T1c	14 (22.6)	52 (24.1)	
T2	15 (24.2)	89 (41.2)	
T3/T4	4 (6.5)	19 (8.8)	
Unknown tumor size	12 (19.4)	37 (17.1)	
Nodal Status* – *n*, (%)			0.02
N0	54 (87.1)	156 (72.2)	
N1/N2	8 (12.9)	60 (27.8)	
Biologic Subtype* – *n*, (%)			0.37
ER + HER2-	29 (46.8)	105 (48.6)	
TNBC	32 (51.6)	99 (45.8)	
HER2+	1 (1.6)	12 (5.6)	
Treatment Approach – *n*, (%)			0.17
Primary/upfront surgery	51 (70.8)	147 (62.0)	
Neoadjuvant systemic therapy	21 (29.2)	90 (38.0)	
Index Breast Surgery – *n*, (%)			< 0.001
Breast conserving surgery	21 (29.6)	126 (54.1)	
Unilateral mastectomy	10 (14.1)	38 (16.3)	
Bilateral mastectomy	40 (56.3)	69 (29.6)	
Adjuvant Radiation – *n*, (%)			< 0.001
Yes	25 (34.7)	149 (62.9)	
No	47 (65.3)	88 (37.1)	
Adjuvant Endocrine therapy – *n*, (%)			0.02
Yes	16 (22.2)	87 (36.7)	
No	56 (77.8)	150 (63.3)	
Any Chemotherapy Receipt^†^ – *n*, (%)			0.02
Yes	43 (59.7)	176 (74.3)	
No	29 (40.3)	61 (25.7)	

*ER + HER2-* estrogen receptor positive, HER2-negative, *GPV* Germline pathogenic variant, *HER2* + HER2-positive, *MRI* magnetic resonance imaging, *TNBC* Triple negative breast cancer

*Reported for invasive breast cancer cases only

^†^Includes neoadjuvant or adjuvant chemotherapy

Pre-diagnostic awareness was associated with a significantly higher use of bilateral mastectomy at index surgery (56.3% vs. 29.6%, *p* < 0.001) and lower use of adjuvant radiation (34.7% vs. 62.9%, *p* < 0.001). In those with pre-diagnostic awareness, chemotherapy was less likely to be administered for treatment of an index breast cancer (59.7% vs. 74.3%, *p* = 0.02). Adjuvant endocrine therapy (22.2% vs. 36.7%, *p* = 0.02) was also less common in those with pre-diagnostic awareness.

## Discussion

In our study of 309 affected *BRCA1/2* and *PALB2* carriers with operable breast cancer, the receipt of chemotherapy following an index breast cancer diagnosis was 70% overall but varied from 57% in *BRCA2* carriers to upwards of 80% in *BRCA1* and *PALB2* carriers. In the setting of early-stage, T1N0 disease, we found that 79% of *BRCA1* carriers still underwent chemotherapy, largely due to more aggressive tumor biology with TNBC.

The biologic differences between *BRCA1* and *BRCA2-*associated breast cancers have been well established in the literature, with data on *PALB2* continuing to evolve [3, 17, 18]. Germline pathogenic variants in these three genes are associated with a greater than 8-fold odds of TNBC relative to women with sporadic breast cancer [17, 19, 20], although among *BRCA2* and *PALB2* carriers, ER + HER2- breast cancer is still the most common biologic subtype. Data from the BRIDGES study suggest that protein truncating variants in *BRCA2* and *PALB2* are associated with a 17–22% and 11–13% absolute risk of developing low and high-grade ER + HER2- breast cancer, respectively, and a 7–9% lifetime risk of developing TNBC by age 80. In contrast, protein truncating variants in *BRCA1* have a 22% lifetime risk of ER + HER2- breast cancer but a 40% lifetime risk of developing TNBC by age 80 [17]. 

Although we found that *BRCA1*-associated breast cancers were more likely to be early-stage and node-negative at presentation, they were also more likely to receive chemotherapy due to a higher prevalence of TNBC. Current guidelines recommend consideration of chemotherapy for triple-negative tumors greater than 5 mm and endorse its use for all TNBC beyond 1 cm (T1cN0 disease) [5, 21, 22]. The use of chemotherapy – including neoadjuvant chemotherapy– for stage I TNBC has increased over time, reflecting changes in the treatment paradigms for high-risk biologic subtypes [23, 24]. In our subgroup analysis of 89 T1N0 breast cancers, 56% of *BRCA1*-associated cases were TNBC compared to only 18% of *BRCA2* and *PALB2-*associated cases. Although similar treatment patterns were seen within biologic subtypes of T1N0 disease regardless of germline pathogenic variant (85–90% of T1N0 TNBC patients received chemotherapy compared to 52–60% of ER + HER2- breast cancers), the higher prevalence of TNBC in *BRCA1* carriers resulted in 80% of *BRCA1* patients receiving chemotherapy for stage I breast cancer overall. Thus, unaffected *BRCA1* patients who undergo high-risk screening with the goal of early detection should be counselled around the high likelihood of requiring chemotherapy if diagnosed with a breast cancer, even if detected at early stages.

As has been shown in other studies [11, 25, 26], we found that pre-diagnostic awareness of a germline pathogenic variant in *BRCA1*/2 and *PALB2* was associated with a stage-shift towards smaller, node-negative tumors at diagnosis, with a significantly lower proportion receiving chemotherapy (60% vs. 74%) and endocrine therapy (22.2% vs. 36.7%) relative to those undergoing genetic testing after a diagnosis of breast cancer. In a recent study by Hadar et al. that included 105 *BRCA1/2* germline pathogenic variant carriers diagnosed with breast cancer, pre-diagnostic awareness of their *BRCA1/2* status was similarly associated with earlier detection with higher rates of stage 0-I disease (86% vs. 39%, *p* < 0.001) as well as lower rates of chemotherapy receipt (29% vs. 79%, *p* < 0.001) [11]. In another study by Bernstein-Molho et al. of 225 Israeli *BRCA1/2* carriers, chemotherapy was recommended in 51% of known carriers with pre-diagnostic awareness compared to 80% of latent carriers who underwent genetic testing during oncological treatment or follow up (*p* < 0.001) [25]. The method of breast cancer detection was also dramatically different between these two groups, with 58% of known carriers having breast cancer diagnosed by MRI screening, compared to the majority (65%) of latent carriers presenting with a self-detected palpable mass, the latter of which was more likely to be node-positive (48.5% vs. 11.5%, *p* < 0.001). Results from our study show similar patterns of detection, with 61% of known carriers presenting with an MRI-detected abnormality compared to those without awareness of their germline pathogenic variant, of which 70% presented with a clinical abnormality, typically in the form of a palpable mass. It is also notable that no patients with pre-diagnostic awareness in our study presented with stage IV disease. Recent data from the Hereditary Breast Cancer Clinical Study Group support a survival benefit of MRI in unaffected *BRCA1/2* carriers partaking in MRI surveillance programs [2]. In their study of 1756 women, Lubinski et al. found that the risk of breast cancer mortality at 20 years was 3.2% for those undergoing MRI surveillance compared to 14.9% for those who did not. Notably, this appeared to be driven largely by an 80% reduction in breast cancer related deaths in *BRCA1* carriers, whereas there was no statistically significant effect of MRI on reducing mortality in those with *BRCA2* pathogenic variants.

In addition to enabling enhanced surveillance and access to preventive strategies, early awareness of a germline pathogenic variant in *BRCA1/2* or *PALB2* vastly influences surgical decision making when a patient is diagnosed with breast cancer. In the current study, 72 (23.3%) of patients underwent genetic testing prior to their breast cancer diagnosis, whereas 105 (34.0%) had testing and result disclosure after diagnosis but before index breast surgery, and the remaining 132 (42.7%) underwent testing and/or result disclosure after index surgery. The high proportion of patients who were unaware of their germline pathogenic variant at the time of surgery likely contributed to the high breast conserving surgery rate of 48.4%, as those lacking knowledge of their genetics status had a 76% rate of breast conservation compared to only 28.6% in those aware of their carrier status. By contrast, women in our study who were aware of their *BRCA1/2* or *PALB2*-positive status prior to diagnosis underwent bilateral mastectomy as their index breast surgery in 56% of cases.

Similar to other studies [27, 28], we have previously shown that preoperative result disclosure increases uptake of bilateral mastectomy by 70% and reduces the need for adjuvant radiation in a large percentage of carriers who forgo breast conserving treatment for therapeutic mastectomy [29]. Several guidelines currently support consideration of unilateral therapeutic and contralateral risk reducing mastectomy as a treatment option for affected *BRCA1/2* and *PALB2* carriers, although breast conserving therapy remains reasonable for eligible patients who desire this approach [30–32]. As mainstream genetic testing efforts that streamline result disclosure are more widely implemented, better informed local therapy decisions are expected to be cost-effective and will increase the opportunity for surgical prevention [33–35]. 

Data on treatment and chemotherapy receipt in *PALB2* carriers who develop breast cancer remains sparse, in large part because of the more recent discovery and characterization of *PALB2* as a breast cancer susceptibility gene and the relative rarity of pathogenic variants in *PALB2* - seen in only 0.2–0.5% of invasive breast cancers [19, 36] - compared to *BRCA1/2* [4, 14, 37]. Single-institution series from the United States suggest bilateral mastectomy uptake is approximately 60% in *PALB2* carriers [38], while studies from population-based data that combine *PALB2* with other non-*BRCA* carriers such as those with moderate penetrance variants in *ATM* and *CHEK2* report expectedly lower bilateral mastectomy rates of 43% [39]. In our study, *PALB2* was associated with a 37% likelihood of bilateral mastectomy at index breast cancer surgery, but this did not account for women undergoing risk reducing surgery during follow-up. The available literature also suggests that *PALB2*-associated breast cancers are biologically aggressive, with TNBC diagnosed in approximately one third of breast cancer cases [4, 37]. In the remaining *PALB2* affected carriers with HR + HER2- breast cancer, higher 21-gene recurrence scores (similar to that of *BRCA2* carriers) have been reported, suggesting that tumor biology and chemotherapy response is likely similar between these groups [6]. In our study, *PALB2* carriers were more likely to require chemotherapy relative to *BRCA2* carriers, however analyses were based on a small number of only 19 *PALB2*-cases and were likely underpowered; therefore, further data on this subgroup are needed.

Our study has several additional limitations related to its retrospective cohort design and prolonged period of study between 2002 and 2022, during which indications for chemotherapy were changing. Furthermore, we lacked information on the rationale for chemotherapy decisions by treating oncologists, and as stated above, the *PALB2* subgroup was limited. For the patients with pre-diagnostic awareness, we did not collect information on long-term adherence to high-risk MRI screening, nor did we have information on participation and adherence to provincial mammographic screening programs, which begin at 50 years old in Canada, for those with post-diagnostic awareness. Despite the stated limitations, our study is one of the first to evaluate *PALB2* germline pathogenic variant carriers and to our knowledge is one of the largest in the reported literature to address the topic of chemotherapy receipt in those with pre-diagnostic awareness of their germline pathogenic variant status.

Our results suggest a 55–80% likelihood of receiving chemotherapy for *BRCA1/2* breast cancer and provide early estimates for patients with *PALB2*-associated disease. Furthermore, while these data support the value of pre-diagnostic awareness and high-risk screening to detect cancers early and reduce the need for chemotherapy, they also suggest that *BRCA1/2* and *PALB2* carriers should be counseled around the greater than 60% likelihood of requiring chemotherapy even if diagnosed with early-stage disease. Decision tools and counseling that extend beyond survival outcomes and incorporate estimates around the need for chemotherapy will remain important for unaffected carriers considering preventive options in the future. Additional studies that evaluate the interaction between premenopausal risk reducing salpingo-oophorectomy, hormone-replacement therapy, and endocrine prevention on subsequent breast cancer development, tumor biology, and chemotherapy receipt would also be valuable for women considering risk-management strategies.

## Supplementary Information

Supplementary Material 1.

## Data Availability

The data used during the current study are available from the corresponding author on reasonable request and with institutional review board approval.

## References

[1] Warner E, Hill K, Causer P, et al. Prospective study of breast cancer incidence in women with a BRCA1 or BRCA2 mutation under surveillance with and without magnetic resonance imaging. J Clin Oncol. 2011;1(13):1664–9. 10.1200/JCO.2009.27.0835. 10.1200/JCO.2009.27.0835 PMC4874196 21444874

[2] Lubinski J, Kotsopoulos J, Moller P, et al. MRI surveillance and breast Cancer mortality in women with BRCA1 and BRCA2 sequence variations. JAMA Oncol. 2024;1(4):493–9. 10.1001/jamaoncol.2023.6944. 10.1001/jamaoncol.2023.6944 PMC10905376 38421676

[3] Mavaddat N, Barrowdale D, Andrulis IL, et al. Pathology of breast and ovarian cancers among BRCA1 and BRCA2 mutation carriers: results from the consortium of investigators of modifiers of BRCA1/2 (CIMBA). Cancer Epidemiol Biomarkers Prev. 2012;21(1):134–47. 10.1158/1055-9965.EPI-11-0775. 22144499 10.1158/1055-9965.EPI-11-0775 PMC3272407

[4] Antoniou AC, Foulkes WD, Tischkowitz M. Breast-cancer risk in families with mutations in PALB2. N Engl J Med. 2014;23(17):1651–2. 10.1056/NEJMc1410673. 10.1056/NEJMc1410673 25337756

[5] Gradishar WJ, Moran MS, Abraham J, et al. NCCN Guidelines(R) insights: breast cancer, version 4.2023. J Natl Compr Canc Netw. 2023;21(6):594–608. 10.6004/jnccn.2023.0031. 37308117 10.6004/jnccn.2023.0031

[6] Kurian AW, Ward KC, Abrahamse P, Hamilton AS, Katz SJ. Predicted chemotherapy benefit for breast Cancer patients with germline pathogenic variants in Cancer susceptibility genes. JNCI Cancer Spectr. 2021;5(1). 10.1093/jncics/pkaa083. 10.1093/jncics/pkaa083 PMC7785044 33426465

[7] Layman RM, Lin H, Gutierrez Barrera AM, Karuturi MS, Yam C, Arun BK. Clinical outcomes and oncotype DX breast recurrence Score(R) in early-stage BRCA-associated hormone receptor-positive breast cancer. Cancer Med. 2022;11(6):1474–83. 10.1002/cam4.4566. 35128817 10.1002/cam4.4566 PMC8921901

[8] Shah PD, Patil S, Dickler MN, Offit K, Hudis CA, Robson ME. Twenty-one-gene recurrence score assay in BRCA-associated versus sporadic breast cancers: differences based on germline mutation status. Cancer. 2016;15(8):1178–84. 10.1002/cncr.29903. 10.1002/cncr.29903 26859126

[9] Heemskerk-Gerritsen BAM, Jager A, Koppert LB, et al. Survival after bilateral risk-reducing mastectomy in healthy BRCA1 and BRCA2 mutation carriers. Breast Cancer Res Treat. 2019;177(3):723–33. 10.1007/s10549-019-05345-2. 31302855 10.1007/s10549-019-05345-2 PMC6745043

[10] Garstka M, Henriquez A, Kelly BN, et al. How protective are Nipple-Sparing prophylactic mastectomies in BRCA1 and BRCA2 mutation carriers?? Ann Surg Oncol. 2021;28(10):5657–62. 10.1245/s10434-021-10445-9. 34296361 10.1245/s10434-021-10445-9

[11] Hadar T, Mor P, Amit G, et al. Presymptomatic awareness of germline pathogenic BRCA variants and associated outcomes in women with breast Cancer. JAMA Oncol. 2020;1(9):1460–3. 10.1001/jamaoncol.2020.2059. 10.1001/jamaoncol.2020.2059 PMC7349080 32644100

[12] Lambertini M, Ceppi M, Hamy AS, et al. Clinical behavior and outcomes of breast cancer in young women with germline BRCA pathogenic variants. NPJ Breast Cancer. 2021;12(1):16. 10.1038/s41523-021-00224-w. 10.1038/s41523-021-00224-w PMC7880991 33579978

[13] Copson ER, Maishman TC, Tapper WJ, et al. Germline BRCA mutation and outcome in young-onset breast cancer (POSH): a prospective cohort study. Lancet Oncol. 2018;19(2):169–80. 10.1016/S1470-2045(17)30891-4. 29337092 10.1016/S1470-2045(17)30891-4 PMC5805863

[14] Tischkowitz M, Balmana J, Foulkes WD, et al. Management of individuals with germline variants in PALB2: a clinical practice resource of the American college of medical genetics and genomics (ACMG). Genet Med. 2021;23(8):1416–23. 10.1038/s41436-021-01151-8. 33976419 10.1038/s41436-021-01151-8

[15] Isaac D, Karapetyan L, Tamkus D. Association of germline PALB2 mutation and response to Platinum-Based chemotherapy in metastatic breast cancer: A case series. JCO Precis Oncol. 2018;2:1–5. 10.1200/PO.17.00258. 35135128 10.1200/PO.17.00258

[16] Harris PA, Taylor R, Minor BL, et al. The REDCap consortium: Building an international community of software platform partners. J Biomed Inf. 2019;95:103208. 10.1016/j.jbi.2019.103208. 10.1016/j.jbi.2019.103208 PMC7254481 31078660

[17] Breast Cancer Association C, Mavaddat N, Dorling L, et al. Pathology of tumors associated with pathogenic germline variants in 9 breast Cancer susceptibility genes. JAMA Oncol. 2022;1(3):e216744. 10.1001/jamaoncol.2021.6744. 10.1001/jamaoncol.2021.6744 PMC8796069 35084436

[18] Yang X, Leslie G, Doroszuk A, et al. Cancer risks associated with germline PALB2 pathogenic variants: an international study of 524 families. J Clin Oncol Mar. 2020;1(7):674–85. 10.1200/JCO.19.01907. 10.1200/JCO.19.01907 PMC7049229 31841383

[19] Hu C, Hart SN, Gnanaolivu R, et al. A Population-Based study of genes previously implicated in breast Cancer. N Engl J Med. 2021;4(5):440–51. 10.1056/NEJMoa2005936. 10.1056/NEJMoa2005936 PMC8127622 33471974

[20] Shimelis H, LaDuca H, Hu C et al. Triple-Negative Breast Cancer Risk Genes Identified by Multigene Hereditary Cancer Panel Testing. J Natl Cancer Inst. 2018;110(8):855–862. 10.1093/jnci/djy106. 30099541 10.1093/jnci/djy106 PMC6093350

[21] Loibl S, Andre F, Bachelot T, et al. Early breast cancer: ESMO clinical practice guideline for diagnosis, treatment and follow-up. Ann Oncol. 2024;35(2):159–82. 10.1016/j.annonc.2023.11.016. 38101773 10.1016/j.annonc.2023.11.016

[22] Burstein HJ, Curigliano G, Loibl S, et al. Estimating the benefits of therapy for early-stage breast cancer: the St. Gallen international consensus guidelines for the primary therapy of early breast cancer 2019. Ann Oncol. 2019;1(10):1541–57. 10.1093/annonc/mdz235. 10.1093/annonc/mdz235 31373601

[23] Tarantino PLP, Teodoro Vallejo C, Freedman RA, Waks AG, Martínez-Sáez O, Garrido-Castro AC, Lynce F, Tayob N, Lin NU, Tolaney SM, Leone JP. Prognosis and trends in chemotherapy use for patients with stage IA triple-negative breast cancer (TNBC): A population-based study. J Clin Oncol. 2023;41(Number 16_suppl). 10.1200/JCO.2023.41.16_suppl.51.

[24] Prakash I, Neely NB, Thomas SM, et al. Utilization of neoadjuvant chemotherapy in high-risk, node-negative early breast cancer. Cancer Med. 2022;11(4):1099–108. 10.1002/cam4.4517. 34989142 10.1002/cam4.4517 PMC8855910

[25] Bernstein-Molho R, Kaufman B, Ben David MA, et al. Breast cancer surveillance for BRCA1/2 mutation carriers - is early detection early enough? Breast. 2020;49:81–6. 10.1016/j.breast.2019.10.012. 31760168 10.1016/j.breast.2019.10.012 PMC7375664

[26] Chereau E, Uzan C, Balleyguier C, et al. Characteristics, treatment, and outcome of breast cancers diagnosed in BRCA1 and BRCA2 gene mutation carriers in intensive screening programs including magnetic resonance imaging. Clin Breast Cancer. 2010;10(2):113–8. 10.3816/CBC.2010.n.022. 20299317 10.3816/CBC.2010.n.022

[27] Chiba A, Hoskin TL, Hallberg EJ, et al. Impact that timing of genetic mutation diagnosis has on surgical decision making and outcome for BRCA1/BRCA2 mutation carriers with breast Cancer. Ann Surg Oncol. 2016;23(10):3232–8. 10.1245/s10434-016-5328-7. 27338744 10.1245/s10434-016-5328-7 PMC5113286

[28] Yadav S, Reeves A, Campian S, Sufka A, Zakalik D. Preoperative genetic testing impacts surgical decision making in BRCA mutation carriers with breast cancer: a retrospective cohort analysis. Hered Cancer Clin Pract. 2017;15:11. 10.1186/s13053-017-0071-z. 28770017 10.1186/s13053-017-0071-z PMC5530488

[29] Apostolova C, Ferroum A, Alhassan B, et al. Timing of genetic testing in BRCA1/2 and PALB2-Associated breast cancer: preoperative result disclosure increases uptake of risk-reducing mastectomy and reduces unnecessary exposure to radiotherapy. Eur J Surg Oncol. 2024;50(6):108324. 10.1016/j.ejso.2024.108324. 38636249 10.1016/j.ejso.2024.108324

[30] Tung NM, Boughey JC, Pierce LJ, et al. Management of hereditary breast cancer: American society of clinical oncology, American society for radiation oncology, and society of surgical oncology guideline. J Clin Oncol. 2020;20(18):2080–106. 10.1200/JCO.20.00299. 10.1200/JCO.20.00299 32243226

[31] Daly MB, Pal T, Maxwell KN, et al. NCCN Guidelines(R) insights: Genetic/Familial High-Risk assessment: breast, ovarian, and pancreatic, version 2.2024. J Natl Compr Canc Netw. 2023;21(10):1000–10. 10.6004/jnccn.2023.0051. 37856201 10.6004/jnccn.2023.0051

[32] Sessa C, Balmana J, Bober SL, et al. Risk reduction and screening of cancer in hereditary breast-ovarian cancer syndromes: ESMO clinical practice guideline. Ann Oncol. 2023;34(1):33–47. 10.1016/j.annonc.2022.10.004. 36307055 10.1016/j.annonc.2022.10.004

[33] Ain Q, Richardson C, Mutebi M, George A, Kemp Z, Rusby JE. Does mainstream BRCA testing affect surgical decision-making in newly-diagnosed breast cancer patients? Breast. 2022;6:67:30–5. 10.1016/j.breast.2022.12.001. 10.1016/j.breast.2022.12.001 PMC9982265 36577271

[34] Sun L, Brentnall A, Patel S, et al. A Cost-effectiveness analysis of multigene testing for all patients with breast Cancer. JAMA Oncol. 2019;3(12):1718–30. 10.1001/jamaoncol.2019.3323. 10.1001/jamaoncol.2019.3323 PMC6777250 31580391

[35] Kemp Z, Turnbull A, Yost S et al. Evaluation of Cancer-Based Criteria for Use in Mainstream BRCA1 and BRCA2 Genetic Testing in Patients With Breast Cancer. JAMA Netw Open. 2019;2(5):e194428. 10.1001/jamanetworkopen.2019.4428. 31125106 10.1001/jamanetworkopen.2019.4428 PMC6632150

[36] Tung N, Lin NU, Kidd J, et al. Frequency of germline mutations in 25 Cancer susceptibility genes in a sequential series of patients with breast Cancer. J Clin Oncol. 2016;1(13):1460–8. 10.1200/JCO.2015.65.0747. 10.1200/JCO.2015.65.0747 PMC4872307 26976419

[37] Cybulski C, Kluzniak W, Huzarski T, et al. Clinical outcomes in women with breast cancer and a PALB2 mutation: a prospective cohort analysis. Lancet Oncol. 2015;16(6):638–44. 10.1016/S1470-2045(15)70142-7. 25959805 10.1016/S1470-2045(15)70142-7

[38] Cragun D, Weidner A, Tezak A, Clouse K, Pal T. Cancer risk management among female BRCA1/2, PALB2, CHEK2, and ATM carriers. Breast Cancer Res Treat. 2020;182(2):421–8. 10.1007/s10549-020-05699-y. 32445176 10.1007/s10549-020-05699-y

[39] Kurian AW, Ward KC, Abrahamse P, et al. Association of germline genetic testing results with locoregional and systemic therapy in patients with breast Cancer. JAMA Oncol. 2020;1(4):e196400. 10.1001/jamaoncol.2019.6400. 10.1001/jamaoncol.2019.6400 PMC7042883 32027353

