# Supplementary material for: Chemotherapy receipt in affected BRCA1/2 and PALB2 carriers with operable breast cancer: the impact of early detection and pre-diagnostic awareness on clinical outcomes and treatment
Source: Hered Cancer Clin Pract. 2025 Apr 24;23:14. doi: 10.1186/s13053-025-00314-x (PMC12020017; doi:10.1186/s13053-025-00314-x)
Supplement: Supplementary file 1 — Supplementary Material 1. [file 13053_2025_314_MOESM1_ESM.docx]

**Supplementary Table**

**Chemotherapy receipt in affected *BRCA1/2* and *PALB2* carriers with operable breast cancer: the impact of early detection and pre-diagnostic awareness on clinical outcomes and treatment (Wong SM, et al.)**

| eTABLE 1 Chemotherapy receipt in stage I invasive breast cancer by germline pathogenic variant and biologic subtype (n=89) | | |
| --- | --- | --- |
| Germline Pathogenic Variant | **T1N0 cases**  ***n*, (% total)** | **Chemotherapy**  **Receipt**  ***n*, (row %)** |
| *BRCA1* (n=160)  ER+HER2-  HER2+  TNBC | **52 (32.5)**  20 (38.5)  3 (5.8)  29 (55.8) | **41 (78.9)**  12 (60.0)  3 (100.0)  26 (89.7) |
| *BRCA2* (n=130)  ER+HER2-  HER2+  TNBC | **32 (24.6)**  26 (81.3)  1 (3.1)  5 (15.6) | **19 (59.4)**  14 (53.9)  1 (100.0)  4 (80.0) |
| *PALB2* (n=19)  ER+HER2-  HER2+  TNBC | **5 (26.3)**  3 (60.0)  -  2 (40.0) | **3 (60.0)**  1 (33.3)  -  2 (100.0) |

***ER+HER2-*** estrogen receptor positive, HER2-negative; ***HER2***+ HER2-positive; ***TNBC*** Triple negative breast cancer.
